# Hydrogen Storage and Release via Carbon Dioxide Hydrogenation to Formate Salts under High‐Pressure Conditions with Ir Complex and Subsequent Formic Acid Dehydrogenation

**DOI:** 10.1002/open.202500032

**Published:** 2025-02-25

**Authors:** Seo Ono, Ryoichi Kanega, Hajime Kawanami

**Affiliations:** ^1^ Interdisciplinary Research Center for Catalytic Chemistry National Institute of Advanced Industrial Science and Technology 1-1-1 Higashi Tsukuba 305-8565 Ibaraki Japan; ^2^ Graduate School of Pure and Applied Science University of Tsukuba 1-1-1 Tennoudai Tsukuba 305-8577 Ibaraki Japan; ^3^ Research Institute for Energy Conservation Department National Institute of Advanced Industrial Science and Technology 1-1-1 Higashi Tsukuba 305-8565 Ibaraki Japan

**Keywords:** carbon dioxide, hydrogen, hydrogenation, supercritical, carrier

## Abstract

To investigate the potential of formic acid (FA) as a hydrogen carrier, we examined hydrogen storage and production through formate salts generated via CO_2_ reduction under supercritical fluid conditions. Formate salts were synthesized using Cp*Ir homogeneous catalysts to reduce CO_2_ under supercritical conditions (CO_2_: 12 MPa; H_2_: 0.5 MPa; total 12.5 MPa), achieving turnover frequency (TOF) of 10,240 h^−1^ and a turnover number (TON) of 20,480 within 2 h at 50 °C. The maximum formate concentration reached 0.81 mol/L after 18 h. The resulting formate salt solution (0.81 mol/L) was subsequently converted into FA (0.50 mol/L) with a 96 % yield by exchange of the cation (K + to H +) using an ion exchange resin. FA was then dehydrogenated to regenerate hydrogen, achieving a FA conversion exceeding 98 %. This process yielded an overall hydrogen recovery rate of over 90 %. These findings demonstrate a viable method for hydrogen storage and regeneration using formate salts as an efficient hydrogen carrier for future energy applications.

## Graphical abstract

We demonstrated the potential of formic acid for efficient hydrogen storage and regeneration through CO₂ reduction followed by dehydrogenation. Using Cp*Ir catalysts with an amino‐substituted bipyridine ligand, we achieved a 96 % yield of formic acid with a formate concentration of 0.81 mol/L under supercritical conditions, followed by dehydrogenation with over 90 % hydrogen recovery.

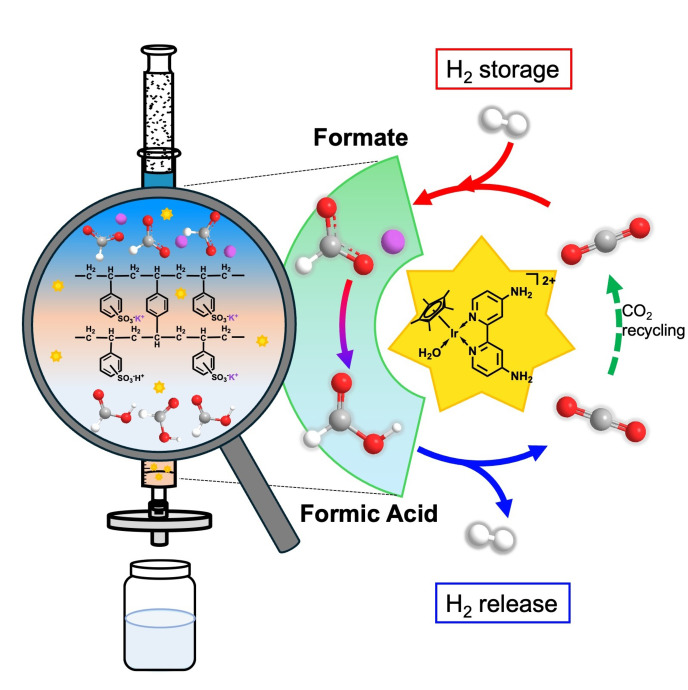

## Introduction

The atmospheric concentration of carbon dioxide (CO_2_) is increasing at an alarming rate, driving critical issues such as global warming, El Niño phenomena, and rising sea levels. To mitigate CO_2_ emissions, global research efforts are focused on three primary strategies: (1) reducing CO_2_ emissions into the atmosphere, (2) capturing CO_2_ from the atmosphere, and (3) achieving carbon recycling by recycling captured CO_2_.^[1]^ Among these strategies, technologies utilizing CO_2_ are indispensable for attaining carbon neutrality, emphasizing the need for continued research and innovation. Within this framework, hydrogen‐based systems that do not emit CO_2_ have emerged as a focal point of attention. However, the low density of the gaseous state of H_2_ necessitates the development of efficient technologies for its storage, transport, and utilization.

**Figure 1:**
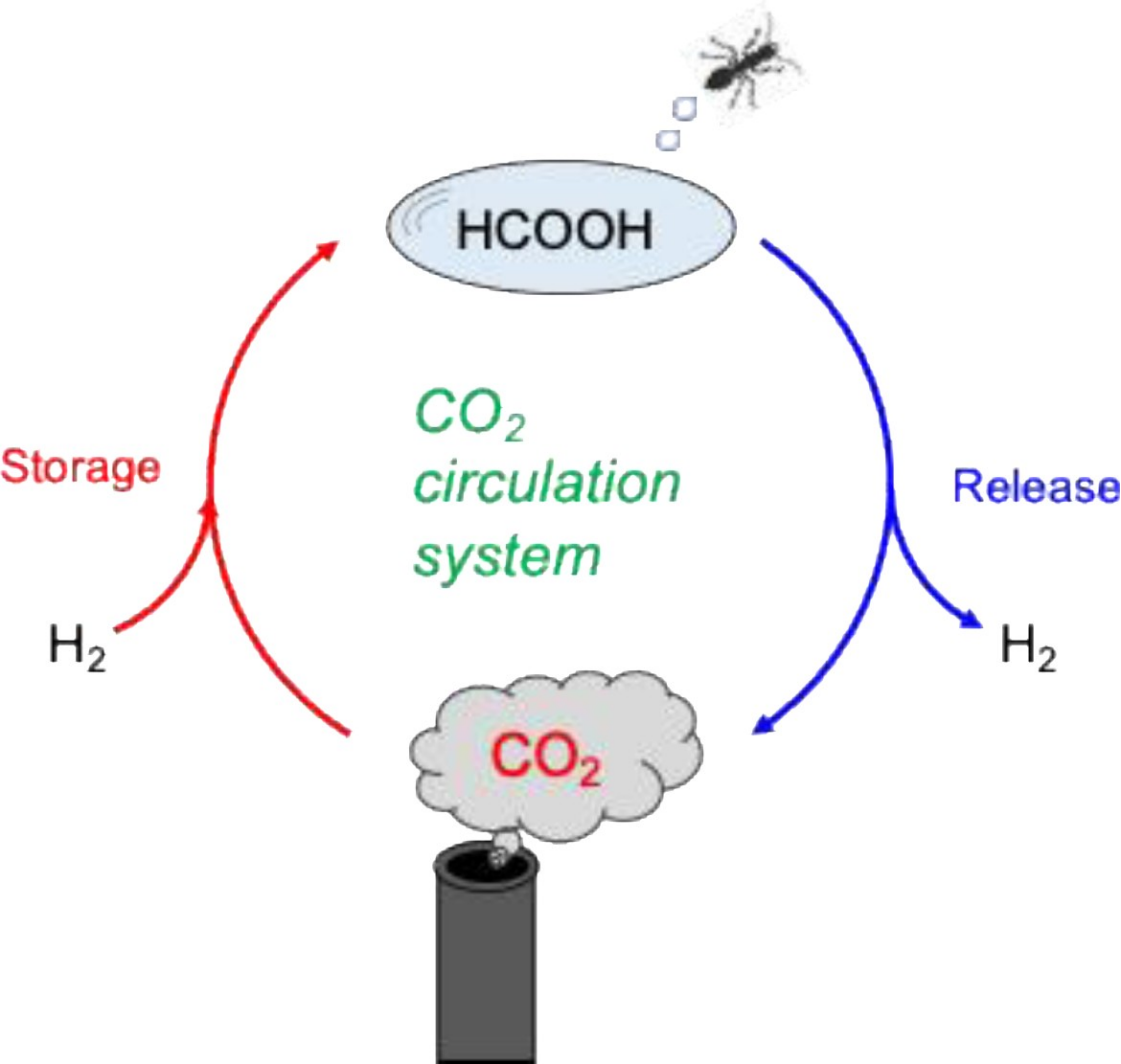
CCHC System with the interconversion between CO_2_ and FA or formate.

In the 1970s, Williams et al. proposed the use of formates as carriers for hydrogen storage and transport,^[2]^ and later, Sasson et al. introduced a similar concept in 1986.^[3]^ Since then, substantial advancements have been achieved in technologies enabling the conversion of CO_2_ into FA and formates. As shown in Figure 1, recent innovations include the development of carbon dioxide circulation hydrogen carrier (CCHC) systems, which utilize the CO_2_‐FA cycle powered by hydrogen generated from renewable energy sources.^[4]^

**Figure 2:**
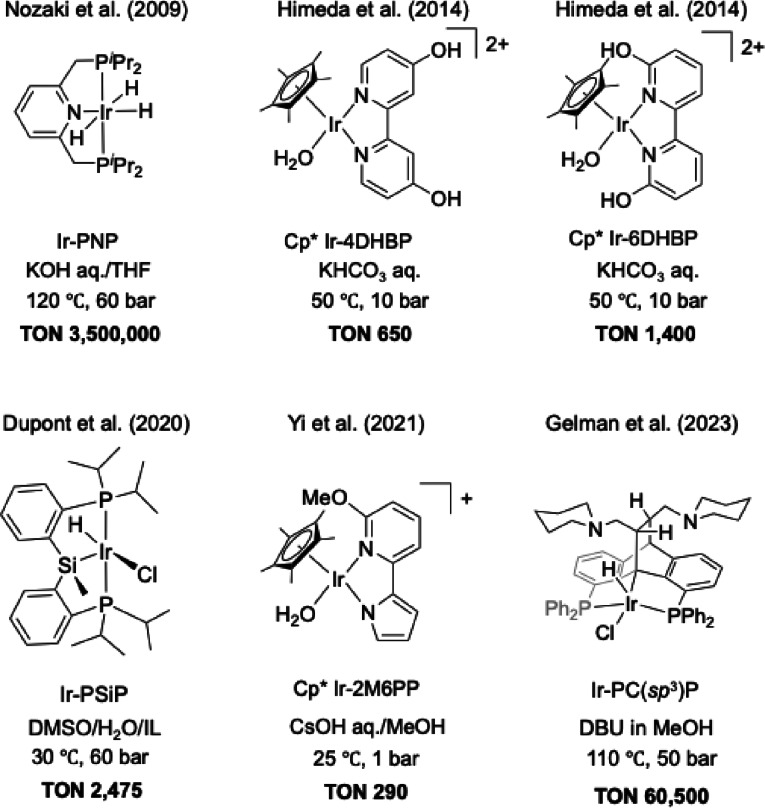
Examples of Ir complexes for CO_2_ hydrogenation.

The critical challenge in CO_2_‐to‐FA conversion lies in the activation of chemically stable CO_2_ and development of catalysts with exceptional activity under mild conditions.^[5]^ Numerous catalysts, particularly homogeneous catalysts such as Ir‐based^[6]^ and Ru‐based complexes,^[7]^ have been extensively investigated for this purpose.^[8]^ Nevertheless, basic conditions are required to thermodynamically drive the reaction. Under these conditions, the direct hydrogenation of CO_2_ leads to the formation of formate, driven by entropy contributions.[^5^, ^6^, ^7^, ^9^] For example, Nozaki *et al*. demonstrated that an Ir‐PNP pincer complex (Figure 2) achieved an impressive turnover number (TON) of 3,500,000 in a KOH aqueous/THF mixed solvent system at 120 °C and 60 bar.^[6a]^ Additionally, Li *et al*. demonstrated the use of a Ru‐CN(H)P complex, which achieved a TON of 169,000 for formate production in CsOH aqueous/THF at 200 °C and 50 bar.^[10]^ A Cp*Ir complex (Cp*: 1,2,3,4,5‐pentamethylcyclopentadienyl) containing a 2,2′‐bipyridine (bpy)‐based ligand exhibited a low turnover frequency (TOF) of 1 h^−1^ for CO_2_ hydrogenation in a 2 M KHCO_3_ aqueous solution at 50 °C and 1 MPa (CO_2_/H_2_ = 1/1).^[11]^ Modifying the bpy ligand by introducing OH groups at the 4‐ and 6‐positions of in Cp*Ir‐4DHBP (4DHBP: 4,4′‐dihydroxy‐2,2′‐bipyridine) and Cp*Ir‐6DHBP (6DHBP: 6,6′‐dihydroxy‐2,2′‐bipyridine) significantly enhanced the TON to 650 and 1400, respectively. Sola *et al*. achieved a TON of 2,475 and produced 0.94 M FA by CO_2_ hydrogenation using an Ir‐PSiP pincer complex (IrClH{κP,P,Si‐Si(Me)(C_6_H_4_‐2‐P^*i*^Pr_2_)_2_}) in [1‐butyl‐2,3‐dimethylimidazolium (BMMIm)][OAc] ionic liquid and DMSO/H_2_O mixed solvent.^[12]^ Yi *et al*. investigated CO_2_ hydrogenation in H_2_O/MeOH mixed solvent with CsOH as a base.^[13]^ They achieved a TON of 290 under ambient conditions using a Cp*Ir complex bearing a proton‐responsive *N*, *N*′‐pyridyl pyrrole ligand. Recently, Gelman *et al*. reported a bifunctional Ir pincer complex with a tertiary amine. In the presence of 1,8‐diazabicyclo[5.4.0]undec‐7‐ene (DBU) and CO_2_ hydrogenation in MeOH solvent, this Ir complex achieved a TON of 60,500 in 72 h at 110 °C.^[14]^

Conversely, numerous catalysts have been developed over time for FA dehydrogenation.^[15]^ The dehydrogenation of FA is preferably conducted under acidic conditions, as FA is one of the strongest organic acids. This reaction can proceed either without any additives or with the addition of formates to adjust acidity.

While numerous reports focus on CO_2_ hydrogenation and FA dehydrogenation, only a limited number of studies demonstrate their continuous integration for hydrogen storage, transport, and regeneration. This is primarily because CO_2_ hydrogenation under basic conditions using homogeneous catalysts is highly efficient; however, the dehydrogenation of formate is significantly slower compared to FA dehydrogenation. As a result, additional steps such as catalyst separation, recovery, and neutralization are required to regenerate FA. To address these challenges, some attempts have been made to simplify catalyst separation by utilizing heterogeneous catalysts for reversible CO_2_ hydrogenation and FA dehydrogenation cycles.^[16]^ Although these reactions can proceed independently, achieving a fully reversible cycle remains a significant challenge.

In contrast, notable successes have been achieved in realizing reversible cycles using homogeneous catalysts. Hull *et al*. demonstrated a reversible system employing a dinuclear Ir complex, where pH adjustment was achieved by adding a base (KHCO_3_) during CO_2_ hydrogenation and an acid (H_2_SO_4_) during FA dehydrogenation.^[17]^ However, the addition of acid resulted in solution dilution and an increase in salt concentration due to neutralization, creating challenges for process scalability. Laurenczy *et al*. developed a reversible system that circumvented the neutralization step by directly dehydrogenating formate within a pressurized H NMR tube.^[18]^ They achieved five reversible cycles with conversion rates of 95 % for CO_2_ hydrogenation and 62 % for formate dehydrogenation. Nonetheless, dehydrogenation under basic conditions remained slow, with a TOF of approximately 6.9 h^−1^. More recently, Beller *et al*. reported the H_2_ storage and regenerate cycles using formate in a triglyme/H_2_O solvent system, employing Ru complexes with PNP pincer ligands as catalysts. This approach achieved TOFs of up to 9,650 h^−1^ with 74 % yield for potassium formate formation and a TOF of 1,858 h^−1^ with 91 % yield for H_2_ regeneration from potassium formate. They successfully repeated these hydrogenation–dehydrogenation cycles in the triglyme/H_2_O solvent system for 40 times.^[19]^

In this context, we previously reported that polyamines strongly interacted with Ir complexes, whereas polyacrylic acid facilitated the adsorption and desorption of iridium complexes depending on their acidity or basicity.^[20]^ Additionally, we demonstrated that ion‐exchange resins effectively converted formate back to FA. Building on these findings, we constructed a CCHC system that integrated CO_2_ hydrogenation, formate transport, and FA dehydrogenation using ion‐exchange resins.

**Figure 3:**
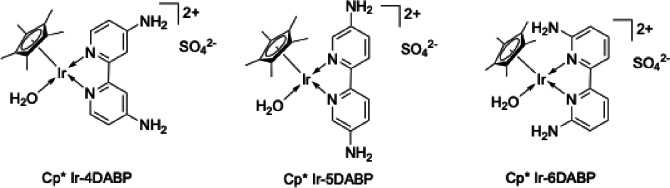
Ir complexes bearing amino‐substituted bipyridine ligand for CO_2_ hydrogenation

**Figure 4:**
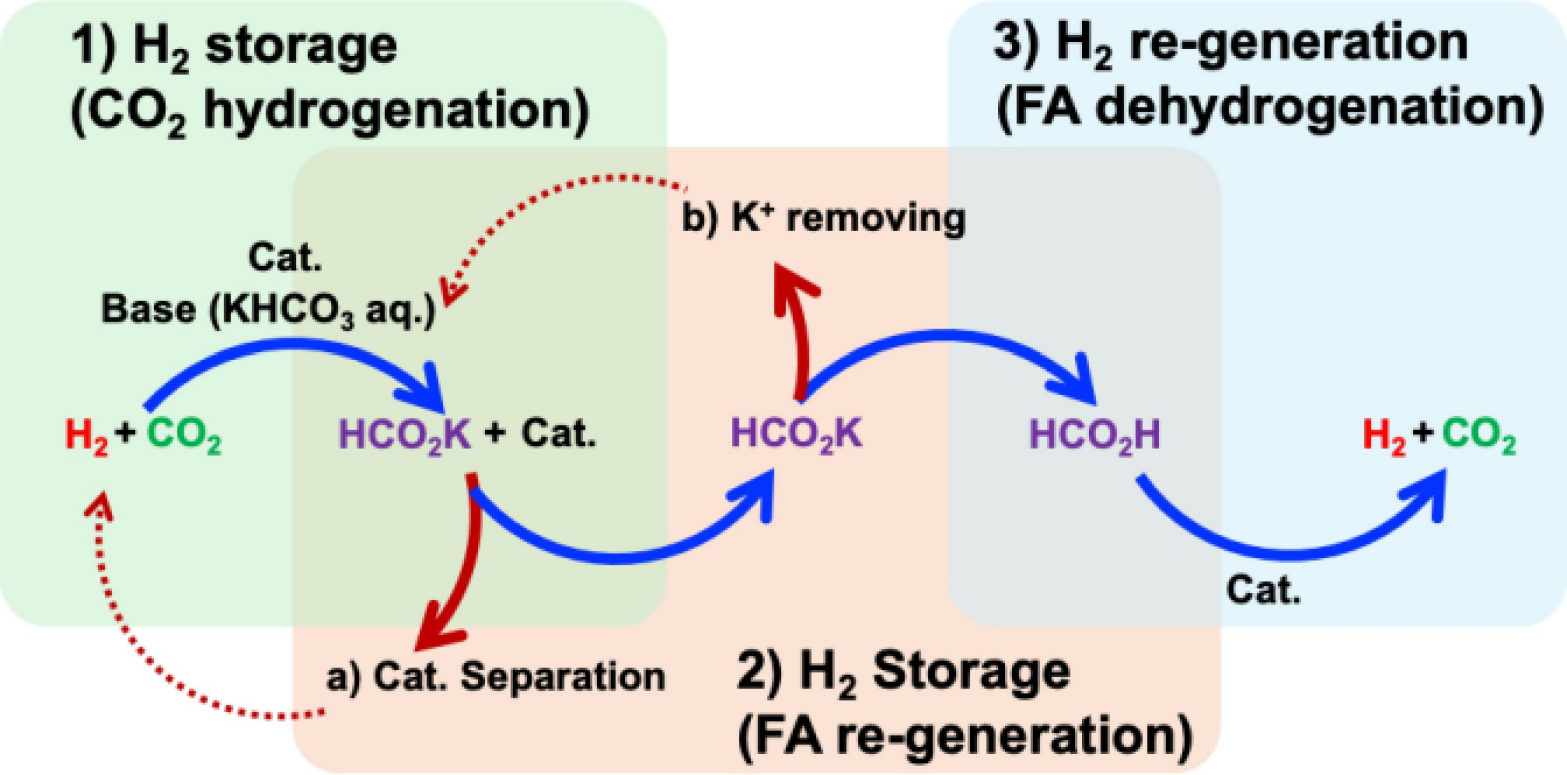
Hydrogen storage through (1) CO_2_ hydrogenation with H_2_ to produce formate salts followed by (2) the conversion of formate into FA, and finally (3) regeneration of H_2_ through the dehydrogenation of the generated FA.

Specifically, we synthesized highly concentrated formate under basic conditions using a Cp*Ir‐4DABP complex (4DABP: 4,4′‐diamino‐2,2′‐bipyridine, Figure 3) under supercritical conditions (Figure 4–1). The resulting formate solution was subjected to catalyst recovery and cation exchange using ion‐exchange resins to regenerate FA (Figure 4–2a, 4–2b), followed by hydrogen release from the regenerated FA (Figure 4–3). This process achieved rapid formate production from CO_2_ and efficient hydrogen regeneration with an overall efficiency exceeding 90 %, as detailed in this report.

## Results and Discussion

### CO_2_ hydrogenation activity of Ir complex under basic conditions

To optimize CO_2_ hydrogenation under basic conditions, we conducted experiments using Cp*Ir−4DABP (0.2 μmol, Figure 3), a catalyst previously shown to exhibit high activity in FA dehydrogenation.^[21]^ The reactions were performed in various basic aqueous solutions (10 mL) within a 30 mL stainless‐steel autoclave. For the initial experiment, a 1 M NaHCO_3_ aqueous solution was used as the reaction medium at 50 °C. CO_2_ and H_2_ were sequentially introduced to achieve a total pressure of 1 MPa (CO_2_: 0.5 MPa, H_2_: 0.5 MPa), and the reaction was allowed to proceed for 2 h. Formate production was analyzed via HPLC, as summarized in Table 1. The results reveal that Cp*Ir−4DABP achieves a TOF of 1,100 h^−1^ and TON of 2,200 after 2 h (entry 1). The para‐substituted amino groups in Cp*Ir−4DABP, a strong electron‐donating group known for its impact on catalyst activity in FA dehydrogenation, also demonstrates exceptional activity in CO_2_ hydrogenation under basic conditions.

**Table 1:** CO_2_ hydrogenation with various basic aqueous solutions (10 mL) with Cp*Ir‐4DABP (0.2 μmol) at 50 °C under 0.5 MPa of H_2_ + 0.5 MPa of CO_2_, reaction time of 2 h.

Run	Base	pH	Formate		
			Conc. / mol L^−1^	TON	TOF / h^−1^
1^[a]^	NaHCO_3_	8.00	0.044	2,200	1,100
2^[a],[b]^	NaHCO_3_	8.00	0.015	760	380
3^[a]^	Na_2_CO_3_	11.72	0.006	330	115
4^[a]^	KHCO_3_	8.10	0.028	1,430	715
5^[a]^	K_2_CO_3_	11.81	0.010	490	245
6^[c]^	CsHCO_3_	8.46	0.047	2,360	1,180
7^[c]^	CsHCO_3_	11.94	0.023	1,170	585

[a] 1 M basic aqueous solutions; [b] Hydrogen was added first. [c] 0.5 M basic aqueous solution.

**Figure 5:**
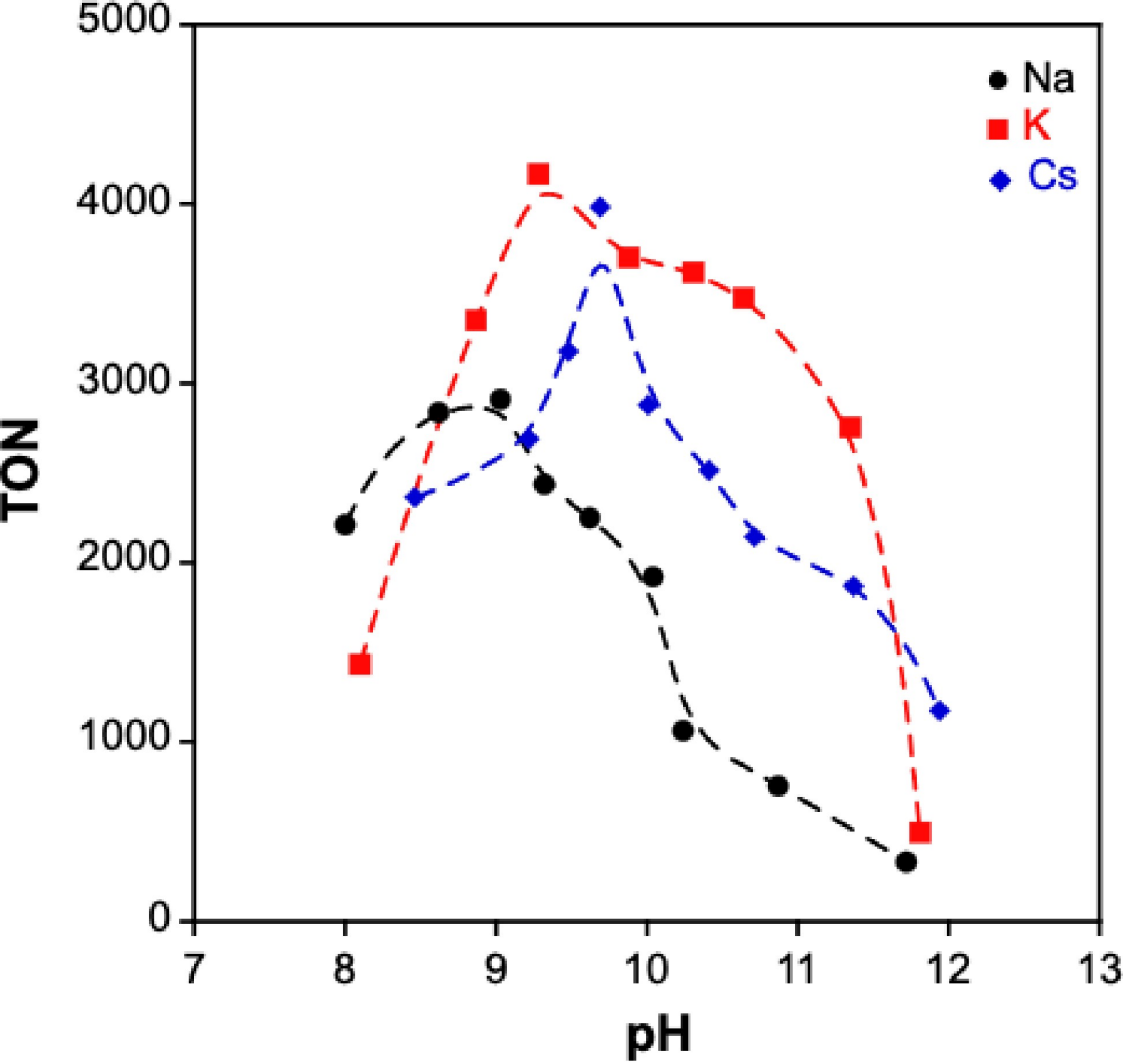
pH dependence of the TON values for CO_2_ hydrogenation under constant 0.5 MPa H_2_ partial pressure.

**Figure 6:**
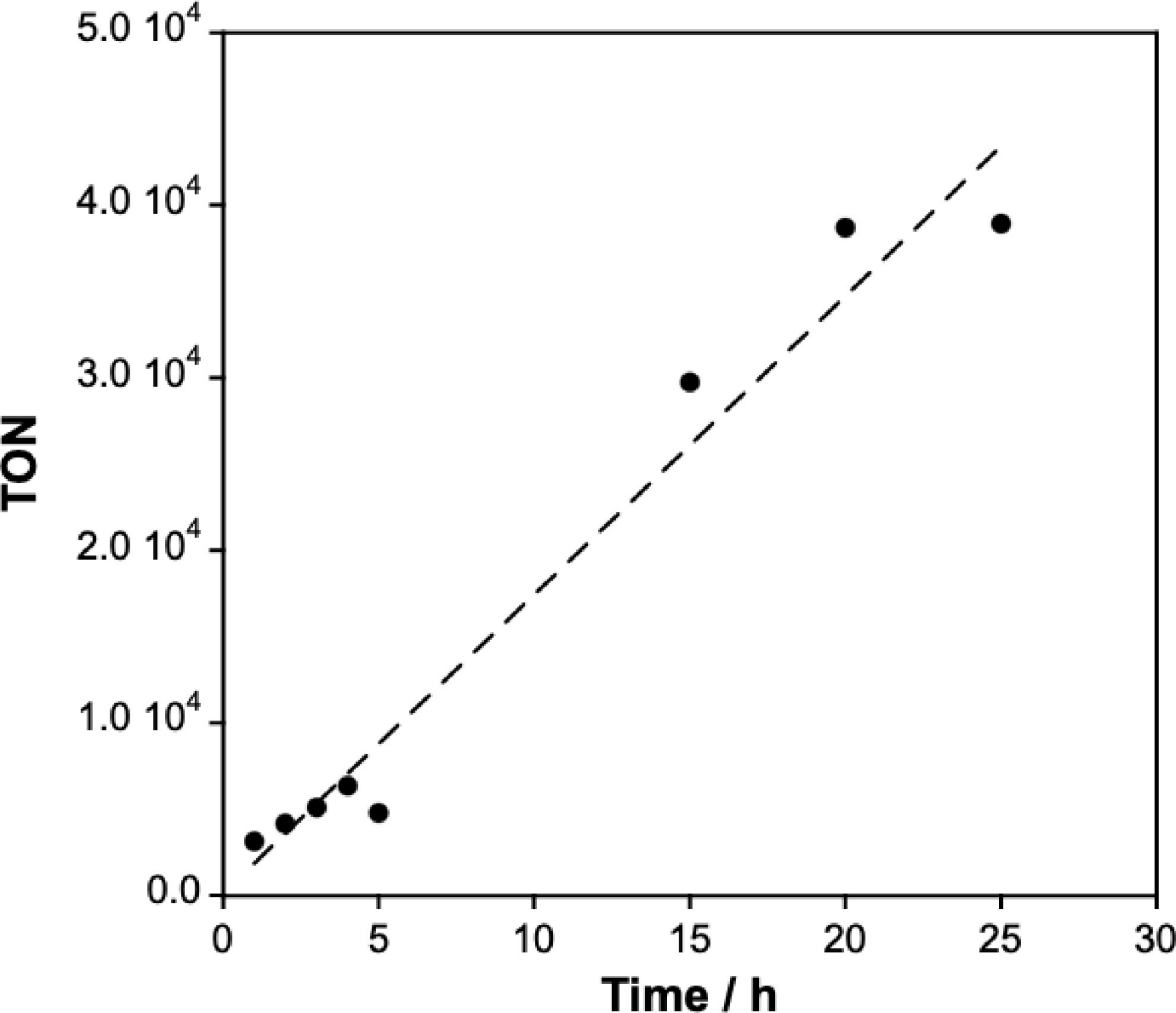
Time course of TON values for CO_2_ hydrogenation.

Subsequently, we examined the effect of different bases on CO_2_ hydrogenation (Table 1). A comparison revealed that bicarbonates of Na, K, and Cs exhibited higher reactivity than their carbonate counterparts, likely due to differences in pH. To further investigate the influence of pH on CO_2_ hydrogenation, we conducted reactions using aqueous solutions prepared with bicarbonates (NaHCO_3_, KHCO_3_, CsHCO_3_) and carbonates (Na_2_CO_3_, K_2_CO_3_, Cs_2_CO_3_). As shown in Figure 5, the pH dependence of CO_2_ hydrogenation using Cp*Ir−4DABP indicates that the TON increases with pH, peaking in the range of pH 9–10. The highest reactivity is observed with a potassium‐based solution at pH 9.28, achieving a TON of 4,160. This performance surpasses that of Cp*Ir−6DHBP (6DHBP: 6,6‐dihydroxy‐2,2’−bipyridine) reported under similar conditions (50 °C, 1 MPa: CO_2_/H_2_=1/1).[^11^, ^22^] Moreover, we evaluated the activity of Cp*Ir−5DABP and Cp*Ir−6DABP complexes, which feature amino group substitutions at the meta and ortho positions, respectively, at pH 9.28. These complexes exhibited lower TON and TOF compared to Cp*Ir−4DABP (Table 2, entries 2–4). The change in pH influences the catalytic structure through the acid‐base equilibrium of the amino groups in the dimethylamino‐2,2’−bipyridine ligand.^[21]^ Under conditions of pH≤8.0, the amino group predominantly exists in the electron‐withdrawing −NH_3_^+^ form, resulting in reduced reactivity. Between pH 8 and 9, both −NH_3_^+^ and −NH_2_ forms coexist during the reaction, while in the range of pH 9–10, the amino group primarily adopts the electron‐donating −NH_2_ form, significantly enhancing reactivity (Figure S4). The decline in reactivity at pH≥10 may be attributed to carbonate precipitation after the reaction, which affects solubility (Figure S5). Additionally, we investigated the time‐dependence of the reaction with Cp*Ir−4DABP (Figure 6). The catalytic activity increased over time, achieving a TON of 38,900 and FA concentration of 0.77 mol L^−1^ after 25 h.

**Table 2:** CO_2_ hydrogenation catalyzed by Cp*Ir complexes.^[a]^

run	Catalyst	pH	TON	TOF/h^−1^	Ref
1^[b]^	Cp*Ir−4DABP	pH 9.28	4,160	2,080	This Work
2^[b]^	Cp*Ir−5DABP	pH 9.28	620	310	This Work
3^[b]^	Cp*Ir−6DABP	pH 9.28	560	280	This Work
4^[c]^	Cp*Ir−4DHBP	pH 8.50	650	650	[11 a]
5^[c]^	Cp*Ir−6DHBP	pH 8.50	1,400	1,400	[11 a]

[a] Reaction conditions: 1 M basic aqueous solutions (10 mL) with Cp*Ir−4DABP complex (0.2 μmol) at 50 °C, 1 MPa of CO_2_/H_2_ (1:1), and reaction time is 2 h; [b] using KHCO_3_ and K_2_CO_3_ adjusted to pH 9.28, [c] using 2 M KHCO_3_.

**Figure 7:**
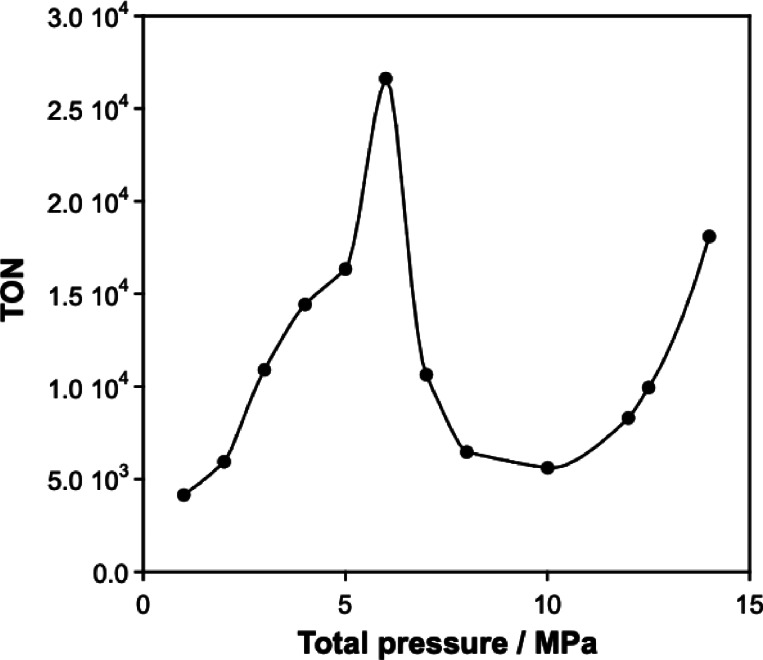
CO_2_ hydrogenation at the various pressures from 1 MPa to 14 MPa with CO_2_ and H_2_ at 1:1 pressure ratio.

**Figure 8:**
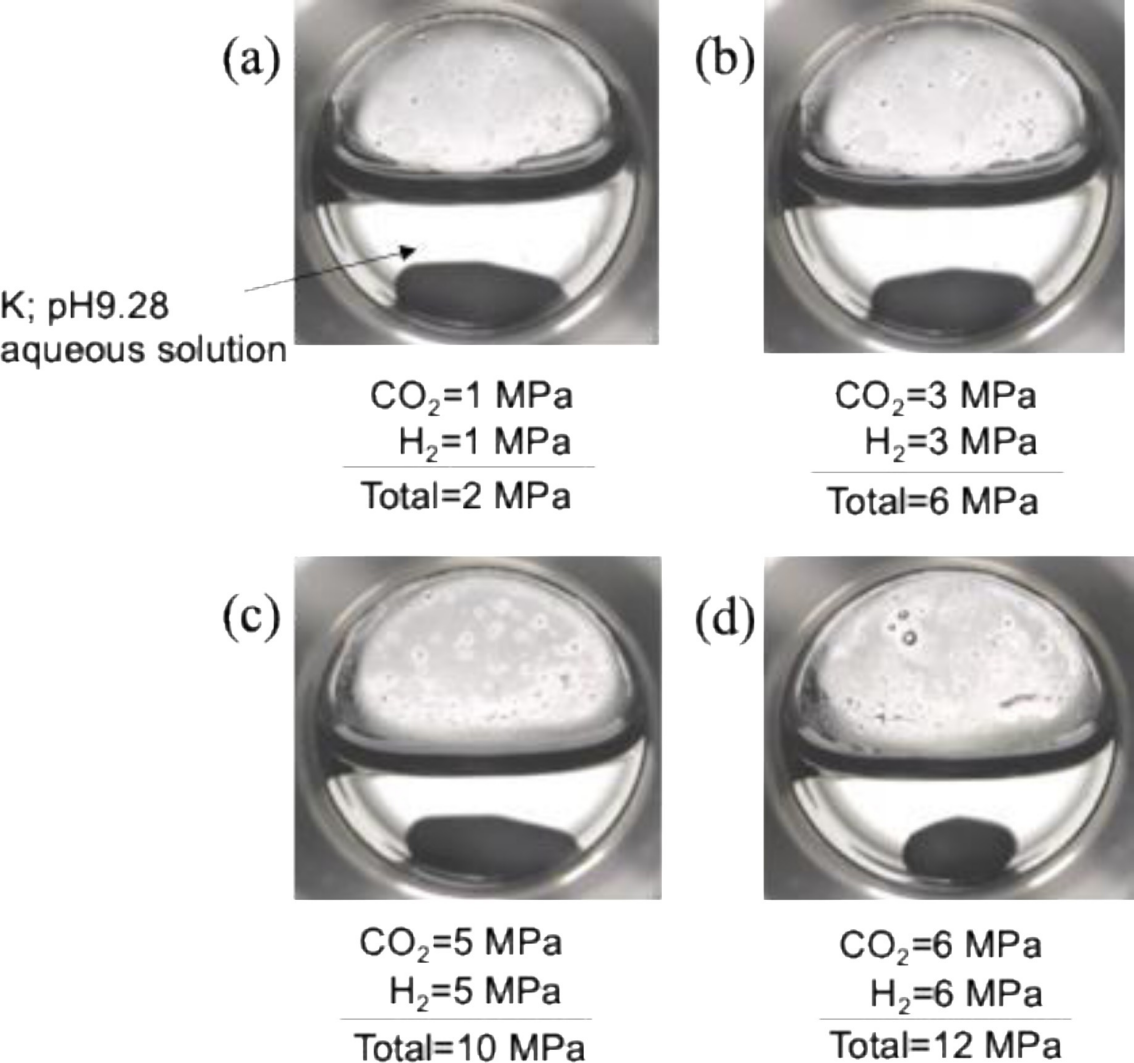
Phase behavior through the view cell in a basic aqueous solution at 50 °C, total pressure of (a) 2, (b) 6, (c) 10, and (d) 12 MPa (H_2_:CO_2_=1:1).

To evaluate reaction efficiency under supercritical conditions, CO_2_ hydrogenation was conducted at high pressures. Initially, CO_2_ was pressurized at room temperature and stabilized at 0.5 MPa, followed by the addition of H_2_ at 0.5 MPa. Subsequently, the CO_2_ pressure was further increased from 0.5 MPa to the desired pressure up to 12 MPa, which is the pressure limitation of the pump for hydrogenation (Figure S6). The TON increased with rising CO_2_ pressure, reaching a maximum TON of 20,480 at 12 MPa. Conversely, when CO_2_ and H_2_ were pressurized in a 1:1 ratio, the behavior differed significantly from the results shown in Figure S6 (Figure 7). In this case, the TON value increased proportionally with pressure, peaking to 26,620 at 6 MPa. At this pressure, the highest formate concentration of 0.53 mol/L was achieved after 2 h (Table S1). However, above 6 MPa, the TON value declined markedly, showing a tendency to increase again beyond 10 MPa. The reaction phase behaviors were monitored using a high‐pressure view cell with sapphire windows (Figure 8). Between 0 MPa and 10 MPa, a biphasic system comprising an aqueous phase and an H_2_+CO_2_ phase was observed. While no visible change occurred in the aqueous phase, beyond 10 MPa, phase separation of the H_2_+CO_2_ phase was observed. It is hypothesized that the mixed gas, predominantly CO_2_ with a smaller fraction of H_2_, transition to a supercritical state beyond approximately 6.5 MPa (Figure 8, including the phase diagram of the H_2_+CO_2_ mixture). Since the catalyst dissolves in water but not in CO_2_, the reaction is confined to the aqueous phase. According to Henry's law, the increased solubility of H_2_ and CO_2_ in water at higher pressures enhances their availability in the aqueous phase, facilitating efficient formate formation through the reaction of H_2_ and CO_2_.

### Dehydrogenation of the Generated Potassium Formate

Given the successful synthesis of a 0.53 mol/L aqueous solution of potassium formate from hydrogen and carbon dioxide under basic conditions, we conducted a proof‐of‐concept experiment to regenerate hydrogen from this potassium formate solution. Moreover, the hydrogen regeneration rate from the hydrogen absorbed as formate was evaluated.

To regenerate FA from the formate solution, the potassium salt must first be removed through neutralization. Two primary approaches for this process include: (1) adding strong acids such as hydrochloric acid or sulfuric acid, followed by FA recovery via distillation or solvent extraction; (2) utilizing membranes and acidifiers to separate FA from formate. For example, Leitner *et al*. described a process in which CO_2_ hydrogenation in a DMSO/*n*‐heptane biphasic system enabled FA extraction into DMSO. The extracted FA was then separated by distillation, with acetic acid as a co‐solvent.^[23]^

**Figure 9:**
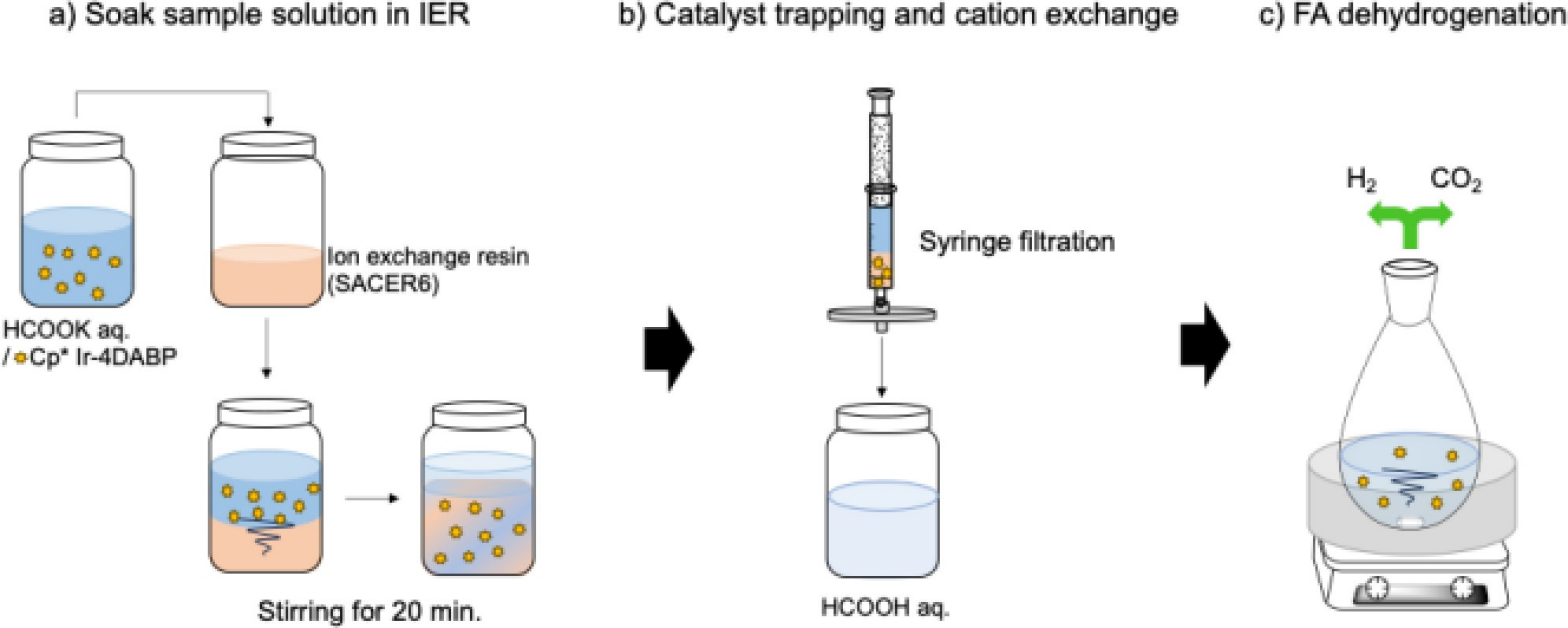
FA and Cp*Ir‐4DABP complex recycle scheme from generated HCOOK solution.

In contrast, to demonstrate a hydrogen storage, transport, and regeneration process without energy‐intensive steps such as distillation, we used ion‐exchange resins to convert potassium formate into FA and conducted experiments to regenerate hydrogen (Figure 9).

The synthesized formate solution contained potassium formate, the Ir catalyst, base, and water. To ultimately generate hydrogen from the resulting “FA” solution, it is necessary to remove the catalyst from the formate solution and exchange the potassium cations with protons. To evaluate the potential for capturing the catalyst using ion exchange resins (IER), 2 μmol of Cp*Ir−4DABP was dissolved in 20 mL of deionized water (Cat.: 0.1 mM) and added to 10 g of various IERs (Figure 9a). The mixture was stirred at room temperature for 20 min and subsequently filtered using a syringe filter (Figure 9b).

**Figure 10:**
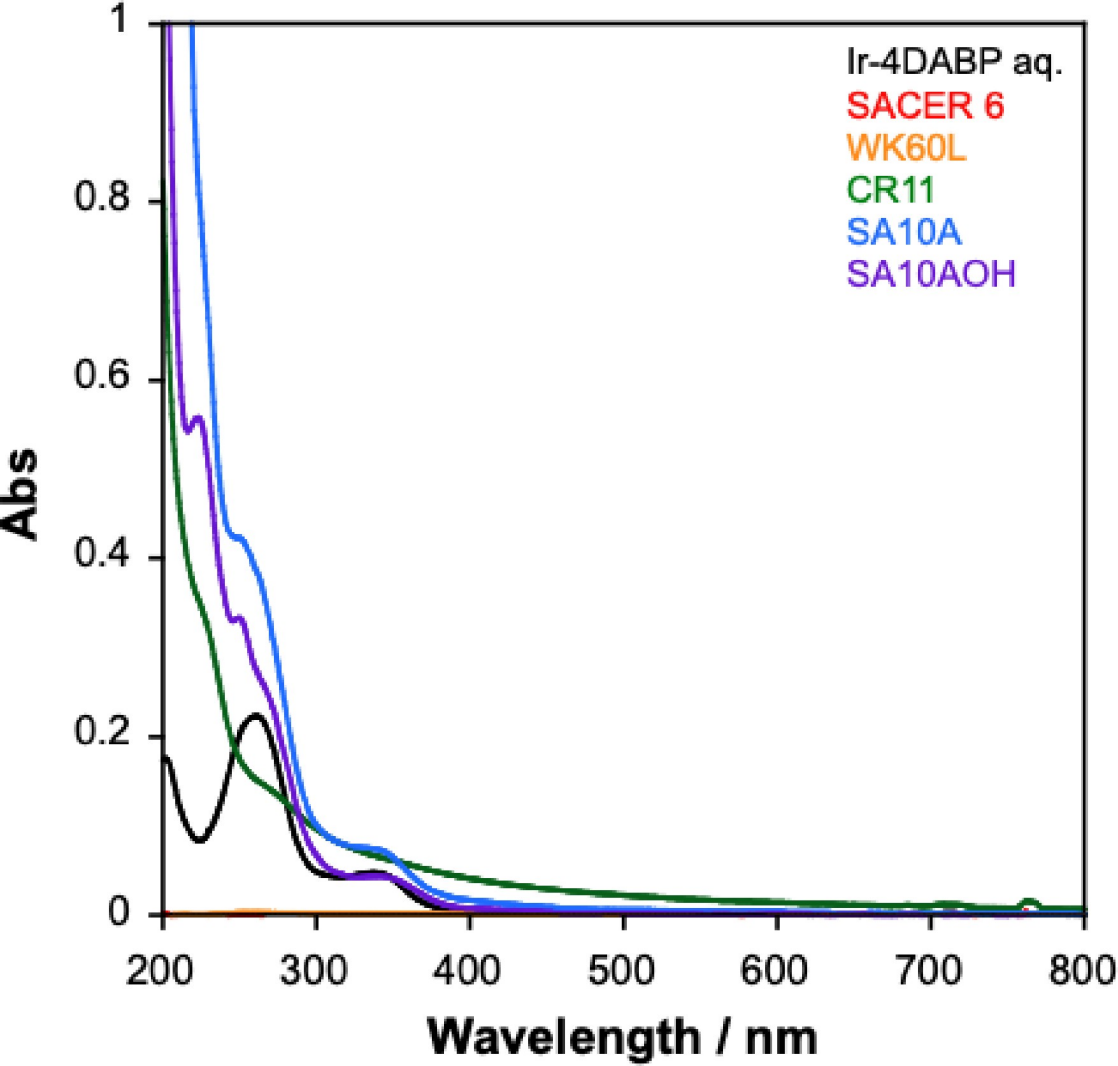
UV‐vis spectra of recovered solution after passing 2 μmol of Cp* Ir‐4DABP aqueous solution through various IERs.

The filtrate was analyzed by UV‐vis spectroscopy to determine whether any Ir complex remained in solution (Figure 10). The UV‐vis spectra of the catalyst solution, after passing through basic IERs such as SA10A and SA10AOH, exhibited no changes, indicating that the catalyst was not adsorbed (Figure S9). In contrast, for acidic IERs such as SACER6 and WK60L, the UV‐vis spectra of the catalyst solution showed no detectable absorption after passing through the IERs, suggesting nearly complete adsorption of the catalyst.

To investigate catalyst elution, 1 M sulfuric acid was added to SACER6, which had absorbed the catalyst. This resulted in the appearance of absorption bands at 345 and 254 nm, indicating catalyst elution from SACER6 (Figure S8). However, impurities from the IER were also eluted (Figure S9), limiting the complete reusability of the catalyst in its current form. Similarly, the CR11 resin adsorbed the catalyst via carboxylic acid functional groups; however, impurities from the resin were also eluted, increasing absorbance at approximately 350 and 250 nm (Figure S9).

Cation exchange tests were conducted using a 1 M HCOOK solution (1.014 M by HPLC) with various IERs; and the results are summarized in Table 3. A 1 M HCOOK solution (20 mL) was stirred with 10 g of IER at room temperature for 20 min and then filtered (Figure 9a and 9b). The pH of the resulting solution was measured, and the concentrations of K^+^ and HCOO^−^ were determined by ion chromatography. When SACER6 was used, the pH of the solution decreased to 1.93, and the K^+^ concentration was reduced to 0.039 mol/L. The HCOO^−^ concentration decreased by approximately 40 %, from 1.014 mol/L (10 mL) to 0.618 mol/L (15.76 mL), which was attributed to dilution by the water content (57.6 %) present in SACER6 (Table S2, Figure S10). The HCOO^−^ recovery rate was calculated to be 96.1 %, indicating that HCOO^−^ was not significantly absorbed by the IER.

**Table 3:** Moisture content of various IERs and K^+^ and HCOO^−^ concentrations after treatment with IERs.

Run	Ion Exchange Resin	Moisture cont. of IER / %	pH	K^+ [a]^		HCOO^−^	
				Conc. / mol L^−1[b]^	Recov. rate / %^[c]^	Conc. / mol L^−1[d]^	Recov. rate / %^[e]^
1	SACER6	57.6	1.93	0.039	6.8	0.618	96.1
2	WK60L	43.5	3.53	0.337	47.7	0.661	93.5
3	CR11	66.6	9.92	0.407	66.8	0.685	99.9
4	SA10A	45.2	7.83	1.086	99.9	0.472	67.6
5	SA10AOH	59.7	14.3	1.247	99.9	0.163	26.9

[a] Initial concentration of HCOOK is 1.014 mol/L; [b] K^+^ concentration (mol L^−1^) after IER absorption test by cation type ion chromatography. [c] Rate of K^+^ recovered; [d] HCOO^−^ concentration (mol L^−1^) after IER absorption test by anion‐type ion chromatography; [e] rate of HCOO^−^ recovered.

For the weakly acidic IER WK60L, the pH decreased from 9.83 to 3.53, and the K^+^ concentration decreased from 1.014 mol/L to 0.337 mol/L, leaving 47.7 % of K^+^ in the solution, indicating incomplete K^+^ exchange. Meanwhile, the HCOO^−^ concentration decreased from 1.014 mol/L to 0.661 mol/L, consistent with the water content of WK60L, yielding an HCOO^−^ recovery rate of 93.5 %.

Using the CR11 resin, 99.9 % of HCOO^−^ was recovered; however, K^+^ adsorption was insufficient, with 66.8 % of K^+^ remaining in the solution after filtration. In contrast, basic IERs such as SA10AOH exhibited minimal K^+^ adsorption and preferentially adsorbed HCOO^−^, with recovery rates of 67.6 % and 26.9 %, respectively. Based on these results, the strongly acidic IER, SACER6, which effectively exchanged K^+^ for H^+^ and achieved a high HCOO^−^ recovery rate, was selected for recycling experiments.

Using 5 g or more of SACER6, the pH of the solution after cation exchange with 20 mL of HCOOK stabilized at 1.95 (Figure S11), and the K^+^ concentration remained constant at 0.01 mol L^−1^ (Figure S12). Additionally, after 20 min, the concentrations of K^+^ and HCOO^−^ anions in the solution remained stable when SACER6 was used (Figure S12)

**Figure 11:**
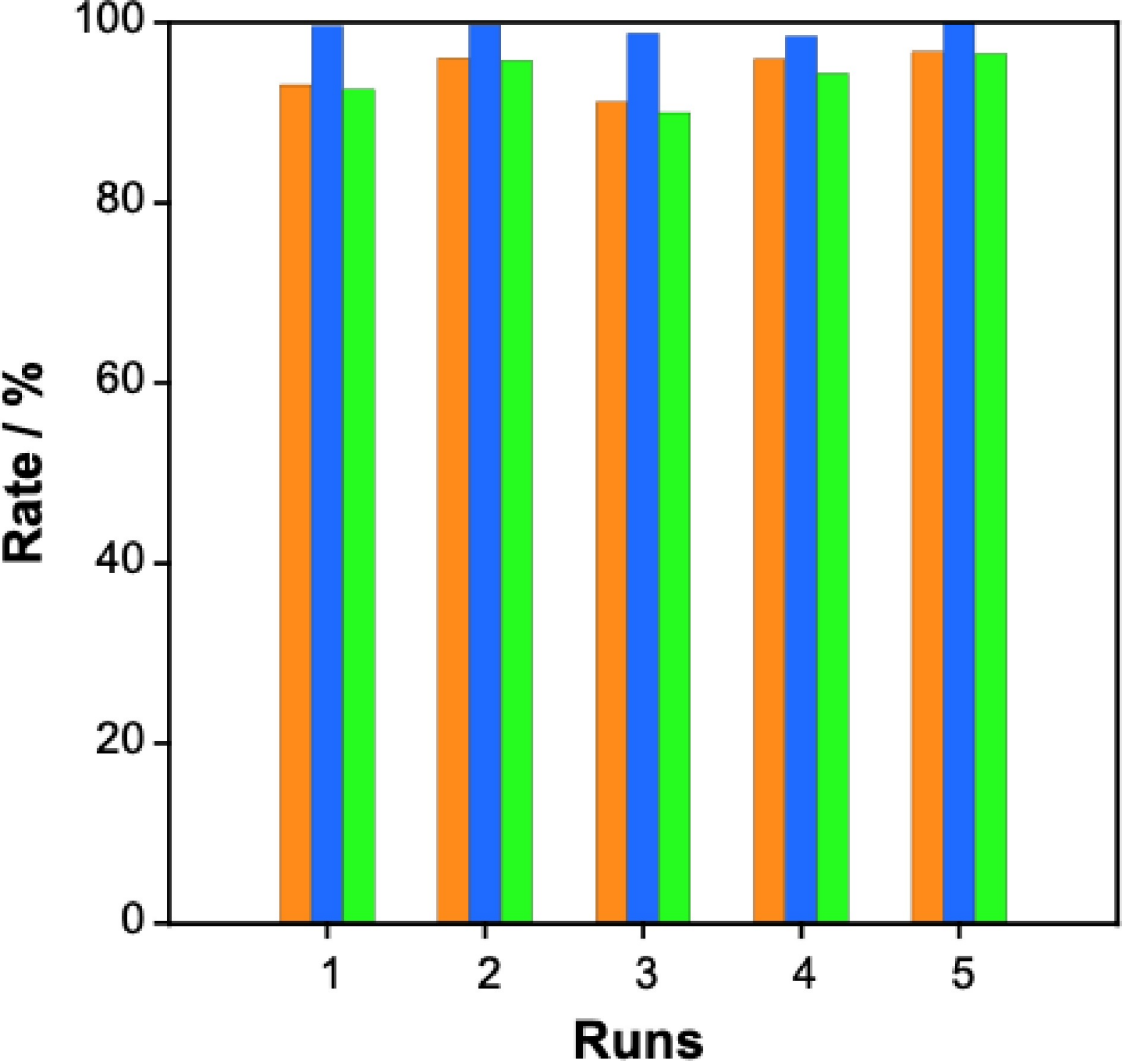
Efficiency of repeated hydrogen storage and regeneration runs via CO_2_ hydrogenation followed by FA dehydrogenation. (1) ▪ Orange bars indicate FA recovery rate, ▪ blue bars indicate FA conversion rate, and ▪ green bars indicate total efficiency of formate to H_2_ conversion.

Finally, a hydrogen regeneration test was repeated 5 times using the potassium formate solution obtained from CO_2_ hydrogenation. The results are summarized in Table S3. A reaction mixture comprising 2 M KHCO_3_ solution (15 mL) and 10 μmol of Cp* Ir‐4DABP was subjected to CO_2_ (6 MPa) and H_2_ (6 MPa) at 50 °C for 18 h. The resulting solution had a pH of 7.6–7.9, with K^+^ and HCOO^−^ concentrations of 2.0–2.4 and 0.53–0.81 mol/L, respectively. Upon treating this potassium formate solution with SACER6 (15 g), the catalyst was adsorbed, and the pH of solution's decreased to 2.1–2.3. This K^+^ concentration was reduced to 0.02–0.10 mol/L, whereas the HCOO^−^ concentration was 0.28–0.50 mol/L. However, each obtained sample solution volume was different (see Table S3). The rate of FA recovery after passing IER was 91.6–96.7 % (orange bars in Figure 11 and Table S3, S4).

Subsequently, Cp* Ir‐4DABP (10 μmol) was added to the obtained FA solution to facilitate FA dehydrogenation for H_2_ regeneration. This process achieved TOFs of 18,580–21,160 h^−1^ (Table S4 and Figure S14), producing 5.38–7.79 mmol of hydrogen with FA conversion rates of 98 % or more for each run (blue bar in Figure 11 and Table S3, S4). H_2_ was successfully regenerated from HCOOK produced by CO_2_ hydrogenation with recovery efficiencies of 90.5–94.6 % (green bar in Figure 11 and Table S3, S4). The gas composition analysis by gas chromatography (TCD detector, detection limit is 10 ppm, Figure S15) confirmed a 1:1 H_2_ to CO_2_ ratio, with no CO detected. Finally, repeating this reaction 5 times resulted in a minimum conversion rate of 90.0 %, highlighting the feasibility and efficiency of the hydrogen storage and regeneration system.

## Conclusion

We demonstrated that potassium formate could be rapidly synthesized using an Ir complex with a bipyridine ligand under basic conditions (potassium bicarbonate solution) in the presence of H_2_ and CO_2_ in a supercritical fluid. However, when the hydrogen and carbon dioxide pressures were maintained at a 1:1 ratio, the formation of potassium formate did not increase proportionally with the total pressure, exhibiting complex behavior. The highest concentration of potassium formate solution (0.8 mol/L) was achieved at total pressures of 6 and 12 MPa.

Furthermore, the potassium formate solution enabled efficient adsorption of the iridium complex catalyst using a strongly acidic IER, which also converted the potassium formate to FA. This regenerated FA was successfully used in a subsequent dehydrogenation catalyzed by an Ir complex (Cp*Ir‐4DABP), achieving over 90.0 % of hydrogen recovery. Additionally, the catalyst‐free formate solution can be stably stored.

These results underscore the potential of this approach for efficient hydrogen storage, transport, and regeneration, offering a sustainable pathway for hydrogen energy system based on FA.

## Conflict of Interests

The authors declare no conflict of interest.

## Supporting information

As a service to our authors and readers, this journal provides supporting information supplied by the authors. Such materials are peer reviewed and may be re‐organized for online delivery, but are not copy‐edited or typeset. Technical support issues arising from supporting information (other than missing files) should be addressed to the authors.

Supporting Information
