# Supplementary material for: Hydrogen Storage and Release via Carbon Dioxide Hydrogenation to Formate Salts under High‐Pressure Conditions with Ir Complex and Subsequent Formic Acid Dehydrogenation
Source: ChemistryOpen. 2025 Feb 25;14(4):e202500032. doi: 10.1002/open.202500032 (PMC11973502; doi:10.1002/open.202500032)
Supplement: Supplementary file 1 — Supporting Information [file OPEN-14-e202500032-s001.pdf]

ChemistryOpen

Supporting Information

Hydrogen Storage and Release via Carbon Dioxide Hydrogenation to Formate Salts under High-Pressure Conditions with Ir Complex and Subsequent Formic Acid Dehydrogenation

Seo Ono, Ryoichi Kanega, and Hajime Kawanami*

Supporting Information

Hydrogen Storage and Release via CO_2 Hydrogenation to Formate Salt with homogeneous Ir complex under High-pressure Conditions and Subsequent Formic Acid Dehydrogenation

*Seo Ono,[1,2] Ryoichi Kanega,[3] Hajime Kawanami[1,2]**

1) *Graduate School of Pure and Applied Science, University of Tsukuba, Tsukuba, 305-8577, Japan.*
2) *Interdisciplinary Research Center for Catalytic Chemistry, National Institute of Advanced Industrial Science and Technology (AIST), Tsukuba, 305-8565, Japan.*
3) *Research Institute for Energy Conservation, National Institute of Advanced Industrial Science and Technology (AIST), Tsukuba, 305-8565, Japan.*

General Procedures

All commercially available chemicals were used received. Sodium bicarbonate ($NaHCO_3$), sodium carbonate (Na_2CO_3), potassium bicarbonate ($KHCO_3$), potassium carbonate (K_2CO_3), cesium bicarbonate ($CsHCO_3$), and cesium carbonate (Cs_2CO_3), Strong acidic cation exchange resin No. 6 (SACER6), Weakly acidic cation exchange resin (Relite™ WK60L), Chelating resin (DIAION™ CR11), Basic anion exchange resin (DIAION™ SA10A, DIAION™ SA10AOH) were purchased from FUJIFILM Wako Pure Chemical Corporation, and $[Cp^*Ir(H_2O)_3]SO_4$ were purchased from Furuya Metal Co., Ltd, and carbon dioxide (99.99%) gas and hydrogen gas (99.99%) was purchased from Tomoe Shokai Co., Ltd. Water was used before deionized by Elix Esen UV3 + Milli-Q Progard TS2 by Merck. All these bases were used as received without further purification for formic acid dehydrogenation (FADH) and CO_2 hydrogenation. Various iridium complex catalysts were synthesized according to previously reported literature, [18] using different bipyridyl (BPY) ligands. The complexes of and various BPY ligands were prepared as described in prior publications.

^1H NMR and ^{13}C NMR spectra were recorded on a Bruker 600 MHz spectrometer (Burker Corp., AVANCE III HD 600 Spectrometer) at room temperature. Chemical shift were given in ppm relative residual solvent in D_2O (^1H＝4.79 ppm) or tetramethylsilane (TMS, ^1H = 0 ppm). Gas Chromatograph (Inficon MicroGC Fusion, Cp-COX column and MS-5A column), Gas Chromatograph equipped with methanizer (Shimadzu GC-2014 + MTN-1 methanizer with ShinCarbon column), Gas Chromatograph equipped Mass Spectrometer (Shimadzu GCMS-QP2010 SE, InertCap FFAP column), HPLC (Agilent Infinity 1260, Shodex RSpak KC-811 column), Ion Chromatograph (Metrohm Eco IC, Cation: TSKgel SuperIC-Cation HS II column, Anion: Metrosep A Supp 4 columm) were used for measuring the products contents and gases contents.

FT-IR spectra were measured directly by ATR methods using FT/IR-6100 (JASCO), and UV-Vis spectra were taken with Cary 60 UV-Vis spectrometer (Agilent Technology Inc.) with a fiber probe. Moisture contents of ion exchange resins (IER) were measured by moisture analyzer (A & D Company ltd., MX-50). The mass analysis was performed using 6230 ESI-TOF-MS system (Agilent Technology Inc.) and ESI-Qq-TOF MS compact (Bruker).

Catalysts Synthesis

Synthesis of Cp*Ir-4DABP

Cp*Ir-4DABP was synthesized according to a previously published method.[18] 4,4'-diamino-2,2'-bipyridine (4-DABP, 93.1 mg, 0.5 mmol) and [Cp*Ir(H_2O)$_3$]SO_4 (238.5 mg, 0.5 mmol) were dissolved in deionized water and stirred at room temperature under N_2 atmosphere for 24 h. The mixture was then filtered and concentrated in vacuum. The resultant yellow precipitate formed after the addition of acetone to the solution was filtered and dried in vacuum at room temperature (257 mg, 0.41 mmol, 82% yield).

Synthesis of Cp*Ir-5DABP

Cp*Ir-5DABP was complexed from ligand and Ir pre-catalyst using the previously published method. 5,5'-diamino-2,2'-bipyridine (5-DABP, 11.7 mg, 0.063 mmol) and [Cp*Ir(H_2O)$_3$]SO_4 (30 mg, 0.063 mmol) were dissolved in deionized water and stirred at room temperature under N_2 atmosphere for 24 h. The mixture was then filtered and concentrated in vacuum. The resultant yellow precipitate formed after the addition of acetone to the solution was filtered and dried in vacuum at room temperature (257 mg, 0.052 mmol, 84 % yield). ^{1}H NMR (600 MHz, D_2O, ppm) δ = 8.39 (d, J = 2.46 Hz, 2H), 7.95 (d, J = 8.94 Hz , 2H), 7.48 (dd, J = 2.40, 8.88 Hz, 2H), 1.63 (s, 15H). ^{13}C NMR (150 MHz, D_2O, ppm) δ = 147.1, 136.9, 125.6, 122.9, 88.8, 88.6, 7.64. ESI-TOF-MS (m/z): [Cp*Ir-5DABP]$^{2+}$ calcd for $C_{20}H_{27}IrN_4$, 257.08; found, 257.07.

Synthesis of Cp*Ir-6DABP

Cp*Ir-6DABP was synthesized according to a previously published method.[18] 6,6'-diamino-2,2'-bipyridine (6-DABP, 39.0 mg, 0.21 mmol) and [Cp*Ir(H_2O)$_3$]SO_4 (100 mg, 0.21 mmol) were dissolved in deionized water and stirred at room temperature under N_2 atmosphere for 24 h. The mixture was then filtered and concentrated in vacuum. The resultant yellow precipitate formed after the addition of acetone to the solution was filtered and dried in vacuum at room temperature (104 mg, 0.16 mmol, 79 % yield).

1.1 Formic acid dehydrogenation

Various 1 M of base (ex: $KHCO_3$ etc.) aqueous solutions were degassed by bubbling with N_2 for 10 min at room temperature. Subsequently, 10 mL of the degassed base solution was transferred to a stainless-steel autoclave. A 40 μL aliquot of a 5 mM of iridium complex aqueous solution (0.2 μmol) was added by syringe. CO_2 was introduced to autoclave made of stainless steel and pressurized up to 2 MPa, and the solution was

stirred at room temperature (1200 rpm) for 90 minutes until the pressure was stabilized, after which the CO_2 pressure was released to 0.5 MPa. H_2 was then pressurized to 0.5 MPa, and the reaction temperature was set to 50 ℃ to increase the reaction temperature. For high-pressure experiments over 1 MPa, H_2 was pressurized to the desired level, followed by re-pressurization of CO_2 using a liquid pump. Pressure and temperature were monitored using a DCS-100A system (Kyowa Electronic Instruments Co., Ltd.). After 2 hours, the autoclave was rapidly cooled in an ice-water bath (to stop the reaction) and slowly returned to atmospheric pressure. The formic acid produced was quantified using high-performance liquid chromatography (HPLC: Agilent Infinity 1260, Shodex RSpak KC-811 column) or ion chromatography (IC: Metrohm Eco IC, Cation: TSKgel SuperIC-Cation HS II column, Anion: Metrosep A Supp 4 column). The presence of by-products, such as CO, was checked using gas chromatography (GC) equipped with a thermal conductivity detector (TCD) and a flame ionization detector (FID).

1.2 Formic acid dehydrogenation

FA aqueous solution passed through IER after CO_2 hydrogenation was placed in a 25 mL round-bottom flask and preheated to 80.0 ℃ (±0.5 ℃) using water bath (EYELA, NTT-2000). Subsequently, Cp*Ir catalyst (6.27 mg, 10 μmol) was added to the FA aqueous solution and the solution was stirred at 1200 rpm. The generated gas was monitored every 2 seconds using gas meter (Shinagawa Co. Ltd., W-NK-0.5A, Japan Flow controls Co. Ltd, MGC-1). Gas volumes were converted to standard temperature and pressure values using the ideal gas law. The generated gas was collected using an aluminum bag directly connected to the gas meter, and gas composition was analyzed using a gas chromatography (GC-TCD). Residual FA after the reaction was investigated using liquid chromatography (HPLC).

1.3 Cation exchange test using various ion exchange resins

The various ion exchange resins (IER) were soaked in pure water and left overnight. The IERs were then collected by filtration and the moisture content was determined by moisture analyzer (MX-50, A & D Instruments Ltd.) (Table 1, Table S2). 10 g of each IER was weighed and transferred into a 50 mL sample bottle. Next, 1 M potassium formate aqueous solution was prepared and 20 mL of this solution was added to each IER.

After 10 minutes of stirring, the sample solution was collected by a plastic syringe with a filter. The pH of each sample solution was measured by pH meter (HORIBA, Ltd., D-52, Standard ToupH Electrode 9615S-10D) and the concentration of formic acid / foramte was analyzed by HPLC and both cationic and anionic IC (Table S2). Results of pH and K^+ concentration showed that SACER6 was the most suitable for cation exchange from K^+ to H^+. The concentration of $HCOO^-$ was less than the adjusted 1 M potassium formate, but this is due to the decrease in concentration caused by the water content of the IER. The $HCOO^-$ concentrations were in close agreement with that obtained from calculations. (Table S2, Figure S10) However, when a basic anion exchange resin was used, the concentration obtained was lower because of the supplementation of anions ($HCOO^-$).

From the concentrations of K^+ and $HCOO^-$, recovery rate (K^+_{recov} and $HCOO^-_{recov}$) was calculated by following method to determine how much K^+ and $HCOO^-$ were present in the recovered solution after each IER was used (see Table 3 in main text).

$$V = (10 + \frac{M_{moist}}{100} \times 10)/1000 \ [L]$$

$$K^+{}_{recov} = 100 - \frac{c_{ini} \times 0.01 - K^+{}_{conc} \times V_{IER}}{c_{ini} \times 0.01} \times 100 \ [\%]$$

$$HCOO^-{}_{recov} = 100 - \frac{c_{ini} \times 0.01 - HCOO^-{}_{conc} \times V_{IER}}{c_{ini} \times 0.01} \times 100 \ [\%]$$

where V_{IER} [L] is the amount of recovered solution considering IER water content, M_{moist} is the water content of the respective IER, c_{ini} is the adjusted initial potassium formate concentration, and K^+_{conc} and $HCOO^-_{conc}$ are the concentrations of K^+ and $HCOO^-$ in the recovered solution, as measured by IC.

Subsequently, 0.1 mM Cp*Ir-4DABP aqueous solution was prepared and 20 mL of solution (2 μmol) was added to each IER weighed 10 g and stirred for 20 min. After collection the sample solution with a syringe filter, those sample were measured by UV-vis Spectrophotometer (Agilent, Cary 60) to confirm the trapping of the catalyst by IER (Figure 10, Figure S9). SACER6 (red line) and WK60L (orange line) showed trapping of the catalyst. In the case of CR11 (green line), no visible catalyst spectrum was observed, but UV absorption of CR11 due to its dissolution in water was observed. For the two basic ion exchange resins (blue and purple lines), catalyst was not captured.

1.4 Strong acidic cation exchange resin No.6 (SACER6) condition test

A screening test for cation exchange was performed using a strong acidic cation exchange resin (SACER6). First, the amount of SACER6 to be used was determined. SACER6 soaked in pure water overnight was filtered and collected. Different amounts of SACER6 were prepared in sample bottles and 10 mL of potassium formate aqueous solution adjusted to 1 M was added. After filtration through a syringe filter, the pH and formic acid / formate concentrations of the resulting solutions were measured by pH meter, HPLC and cationic / anionic IC (Figure S10 to Figure S12). As the SACER6 amount increased, the pH became more acidic and remained almost unchanged at SACER6 amounts of 5 g or more. The concentration of cation K^+ (HCOOK) also tended to decrease with the amount of SACER6. It should be noted that the concentration of the anion $HCOO^-$ (HCOOH + HCOOK) tends to decrease with the amount of SACER6, but $HCOO^-$ is not trapped in SACER6. This decrease in concentration is due to the water contained in SACER6. This was nearly consistent with the concentration obtained from calculations including the moisture content of the SACER6 (Table S2, Figure S10).

Next, changes in the concentration of cation K^+ (HCOOK) with time of soaking in SACAER6 were examined (Figure S13). A little sample was collected after each time step and analyzed by cationic IC. The concentration of cation K^+ (HCOOK) decreased from 5 to 20 minutes after the start of cation exchange and remained constant after 20 minutes.

1.5 CO_2 hydrogenation and FADH recycling test using SACER6

2 M $KHCO_3$ aqueous solution was prepared and degassed via N_2 babbling. After preparing the solution, 15 mL of aqueous solution was transferred into a stainless autoclave (vessel volume is 30 mL) equipped with thermos couple and pressure transducer. Next, 10 μmol of Ir-4DABP was added into the autoclave. Then, CO_2 was added to over 0.5 MPa at room temperature and then the mixture was stirred (1200 rpm) for 2 hours. After reaching the pressure stabilized, CO_2 pressure was adjusted to 0.5 MPa. Subsequently, H_2 was added further 6 MPa, and CO_2 was added until the total pressure reached 12 MPa. Then, increased temperature at 50 °C and the reaction was started. Total pressure and temperature inside the autoclave were monitored during the reaction using data logger (DCS-100A system by Kyowa Electronic Industry Co., Ltd). After 24 hours reaction, the autoclave was cooled with ice water to stop the reaction, and pressure was slowly released to atmospheric pressure. Formate amount was measured using HPLC and IC, and the presence of by-products such as CO was checked by GC.

After measuring the pH of the formed formate sample, the sample solution was slowly added to 15 g of SACER6. The solution was then stirred for 30 minutes and transferred to a syringe filter for filtration (including SACER 6 washing with water). Cation-exchanged solution was collected in the quantities shown in Table S3. After measuring the pH, 0.1 mL of this solution was used for analysis in the cationic / anionic IC.

From the $HCOO^-$ concentration after CO_2 hydrogenation and through IER, FA recovery rate (FA_{recov}) was calculated in the following equation (Table S4).

$$n_{Hstore} = c_{IER} \times V_{sol}\ [mol]$$

$$FA_{recov} = 100 - \frac{c_{CO_2H} \times 0.015 - n_{Hstore}}{c_{CO_2H} \times 0.015} \times 100\ [\%]$$

where n_{Hstore} is the amount of formic acid, c_{IER} is the concentration of $HCOO^-$ after passing through the SACER6, V_{sol} [L] is the amount of recovered solution after passing through the SACER6, c_{CO2H} is the concentration of HCOOK after CO_2 hydrogenation

After cation exchange, sample solution was transferred to a 50 mL Nas flask and warmed to 80 ℃. Then, 10 umol of Cp* Ir-4DABP complex was added and the formic acid dehydrogenation reaction was started. The volume of gas produced was measured with a gas meter (Shinagawa Co. Ltd., W-NK-0.5A), and the remaining solution after the reaction was measured by HPLC. The FA conversion (FA_{conv}) was calculated from the concentration of formic acid before and after FA dehydrogenation by the following method (Table S4).

$$FA_{conv} = 100 - \frac{c_{IER} - c_{FADH}}{c_{IER}} \times 100\ [\%]$$

where c_{FADH} is the concentration of $HCOO^-$ after FA dehydrogenation.

Finally, amount of released H_2 gas (n_{Hrel}) was calculated and H_2 recovery efficiency (η) was determined by comparing the amount of stored H_2 as formate by CO_2 hydrogenation and the amount of released H_2 by FA dehydrogenation (Table S4).

$$n_{Hrel} = \frac{P \times V}{R \times T} \div 2\ [mol]$$

$$\eta = 100 - \frac{n_{Hstore} - n_{Hrel}}{n_{Hstore}} \times 100\ [\%]$$

where *P*, *R*, and *T* represent the atmospheric pressure, the gas content and room temperature, respectively. *V* is the total gas volume by FA dehydrogenation.

Table S1. Reached concentration of formate by CO_2 hydrogenation and TON, TOF values at each pressure of H_2 and CO_2 (1:1).

Total Pressure / MPa	Potassium formate		
	Conc. / mol L^{-1}	TON	TOF / h^{-1}
1	0.08	4,160	2,080
2	0.12	5,940	2,970
3	0.21	10,900	5,450
4	0.28	14,420	7,210
5	0.32	16,350	8,175
6	0.53	26,620	13,310
7	0.21	10,630	5,315
8	0.13	6,470	3,235
10	0.11	5,620	2,810
12	0.16	8,310	4,155
13	0.19	9,940	4,970
14	0.36	18,100	9,050

Reaction conditions: 1 M potassium aqueous solutions (K; 9.28, 10 mL) with Cp*Ir-4DABP (0.2 μmol) at 50 °C, 2 hours, and pressure ration is H_2:CO_2 = 1:1.

Table S2. Comparing $HCOO^-$ concentrations by used instruments and $HCOO^-$ concentrations by calculation considering water content of IERs.

Ion exchange resin	Moisture content / %	Concentration / mol L^{-1}			
		K^+ by cation IC	$HCOO^-$ by anion IC	$HCOO^-$ by HPLC	$HCOO^-$ by calculated
SACER6	57.6	0.039	0.618	0.613	0.633
WK60L	43.5	0.337	0.661	0.670	0.697
CR11	66.6	0.407	0.685	0.714	0.600
SA10A	45.2	1.086	0.472	0.489	0.689
SA10AOH	59.7	1.247	0.163	0.171	0.626

Table S3. Recycle test for CO_2 hydrogenation and FA dehydrogenation using SACER6.

Cycle	CO_2 hydrogenation			Through SACER6				FADH	
	pH	Conc. / mol L^{-1}		pH	Solution volume / mL	Conc. / mol L^{-1}		HCOOH / mmol	H_2 / mol
		K^+	$HCOO^-$			K^+	HCOOH		
1	7.6	2.19	0.48	2.3	22.6	0.026	0.28	6.42	6.05
2	7.8	2.40	0.60	2.1	17.0	0.021	0.31	5.43	5.38
3	7.9	2.07	0.61	2.2	19.4	0.049	0.37	7.99	7.23
4	7.6	2.24	0.74	2.2	18.0	0.024	0.39	7.06	7.05
5	7.9	2.10	0.81	2.1	17.0	0.023	0.50	8.52	7.79

Table S4. Catalytic activity and formic acid recovery/conversion rate and $HCOO^- \rightarrow HCOOH \rightarrow H_2$ conversion efficiency for each cycle.

Cycle	CO_2 hydrogenation		FA dehydrogenation			Efficiency / %
	FA recov. rate / %	TON	FA conv. / %	TON	TOF / h^{-1}	
1	93.0	800	99.5	600	21,160	92.2
2	96.1	1,020	99.6	540	18,580	90.5
3	91.1	1,070	98.8	720	19,820	90.6
4	95.9	1,140	98.4	700	19,150	94.6
5	96.7	1,460	99.8	780	21,150	91.4

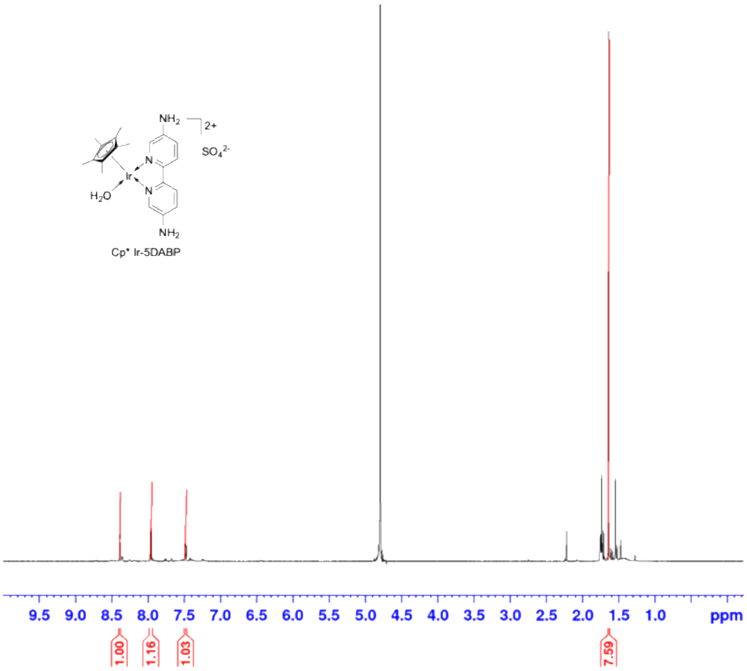

Figure S1. ^{1}H NMR spectra of Cp*Ir-5DABP in D_2O as prepared.

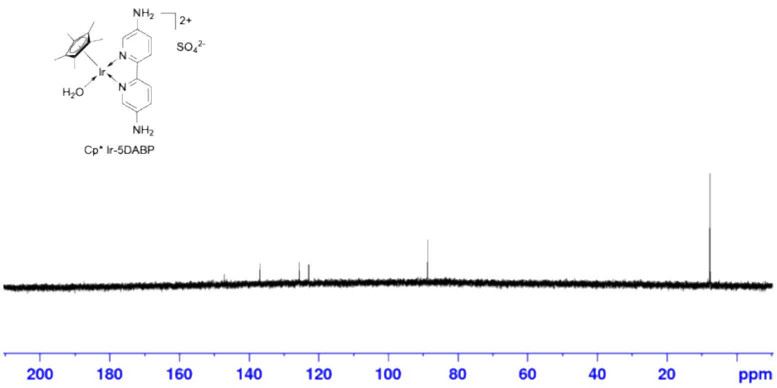

Figure S2. ^{13}C NMR spectra of Cp*Ir-5DABP in D_2O as prepared.

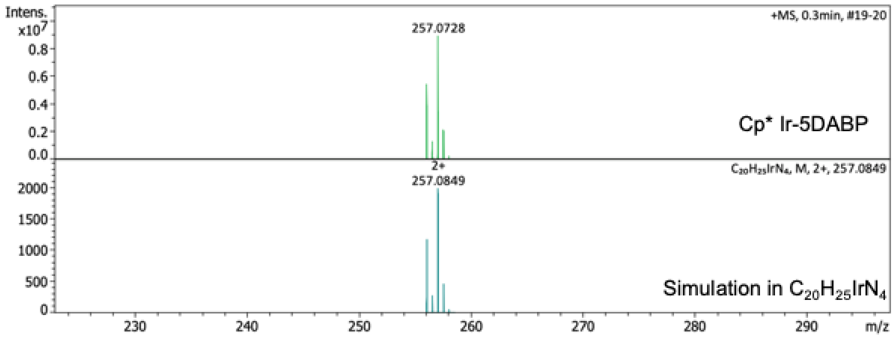

Figure S3. ^{1}H NMR spectra of Cp*Ir-5DABP in D_2O as prepared.

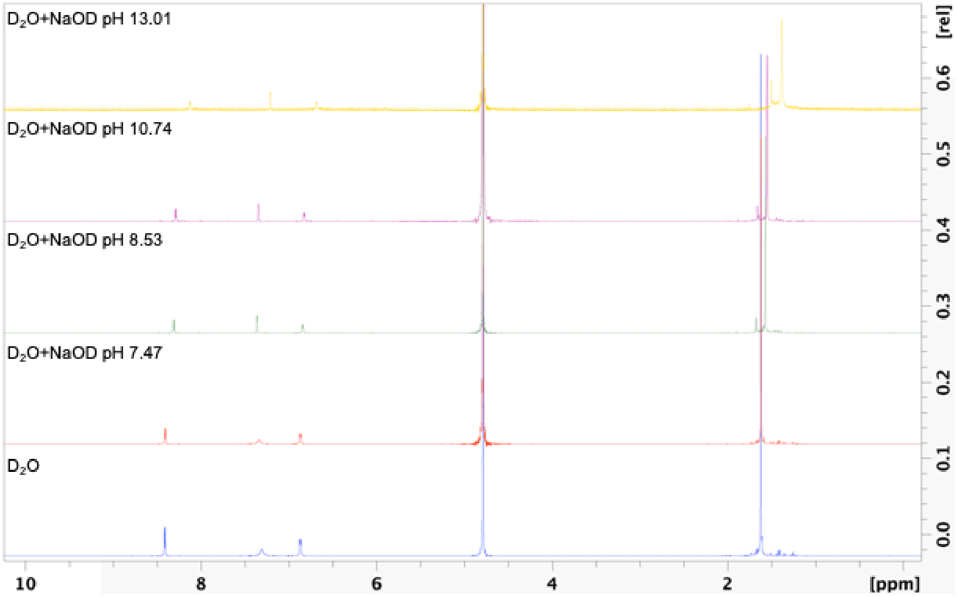

Figure S4. ^{1}H NMR spectra of Cp*Ir-4DABP in D_2O at various pH Values.

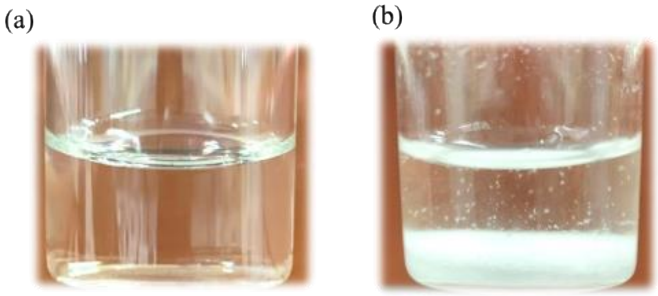

Figure S5. Salt precipitation after CO_2 hydrogenation due to high pH: (a) is the reaction in 1M $NaHCO_3$ aqueous solution, and (b) is the reaction in 1M Na_2CO_3 aqueous solution.

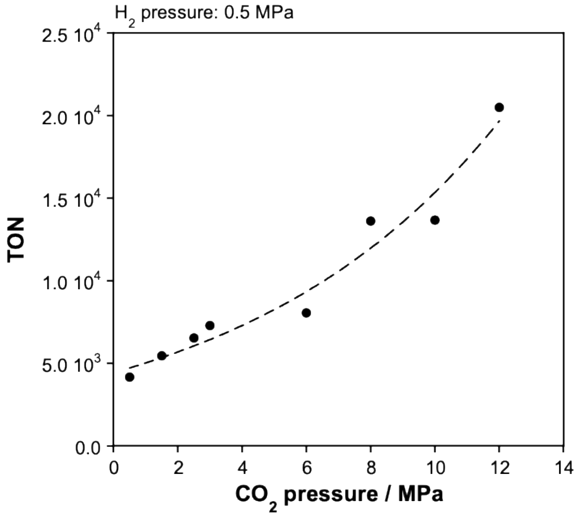

Figure S6. TON as a function of CO_2 pressure for formate production via CO_2 hydrogenation in the K; pH 9.28 aq. solution using Cp*Ir-4DABP (0.2 μmol) at 50 °C.

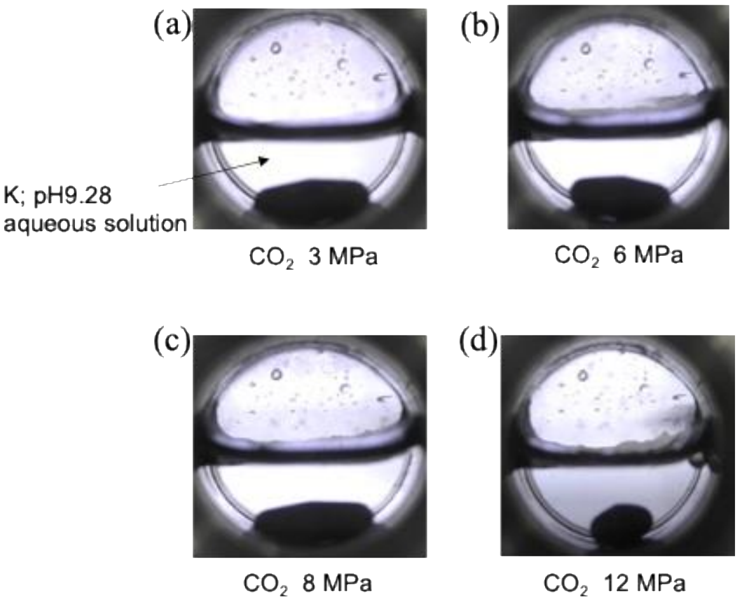

Figure S7. Phase behavior through the view cell in a basic aqueous solution at 50 °C, 0.5 MPa of H_2 and various CO_2 pressure from (a) 3 MPa, (b) 6 MPa, (c) 8 MPa, and (d) 12 MPa.

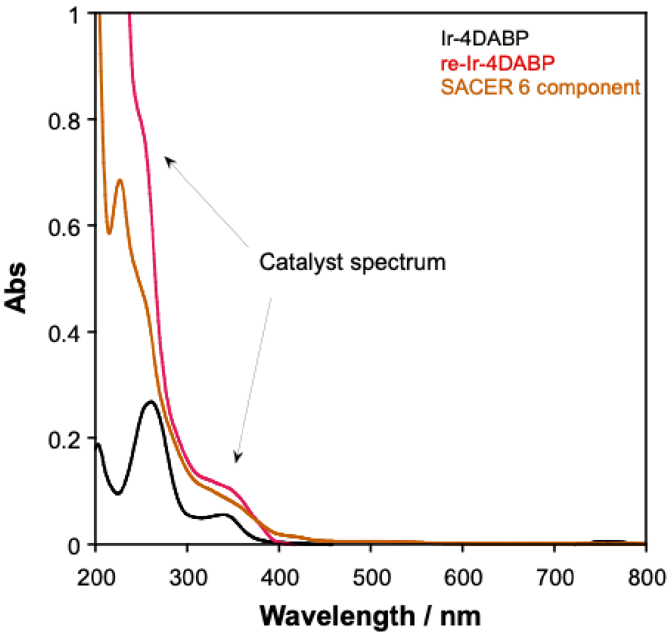

Figure S8. UV-Vis spectra of the solution adding 1M H_2SO_4 aqueous solution after using SACER6.

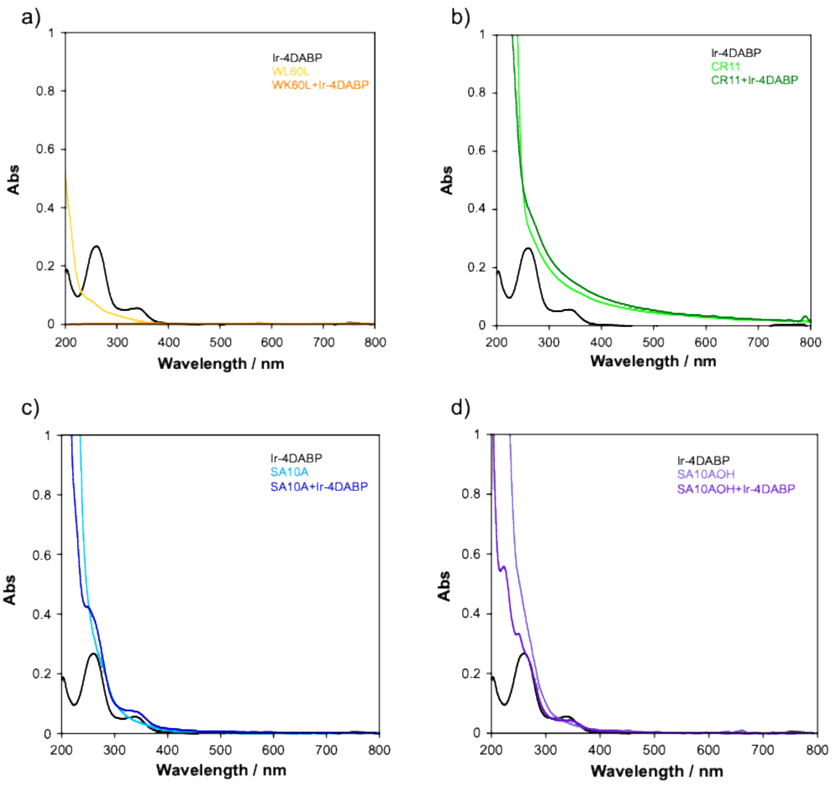

Figure S9 UV-Vis spectra after impurity eluted from WK60L, CR11, SA10A, and SA10AOH in H_2O.

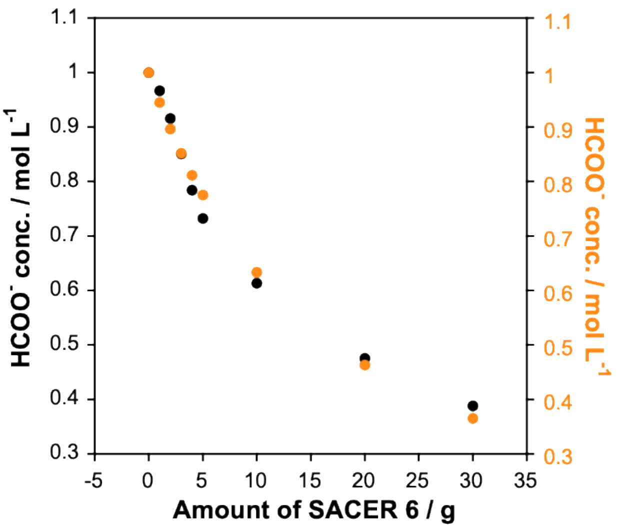

Figure S10. $HCOO^-$ concentration by IC measurement (black axis) and calculated (orange axis) which considering moisture rate after using SACER6.

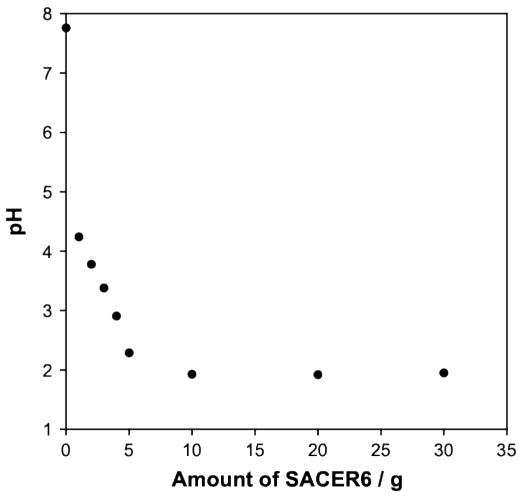

Figure S11. pH dependence on the amount of strong acidic cation exchange resin (SACER6).

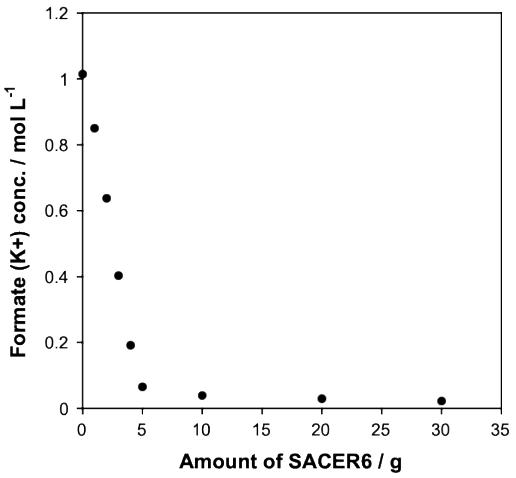

Figure S12. Differences in K^+ concentration with different amount of strong acidic cation exchange resin (SACER6).

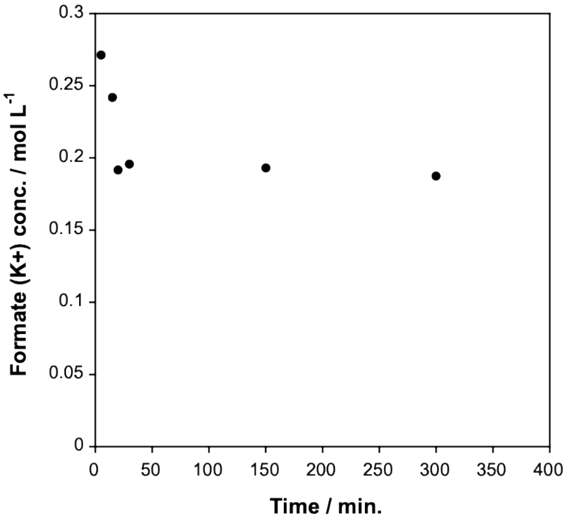

Figure S13. Differences in K^+ concentration with time.

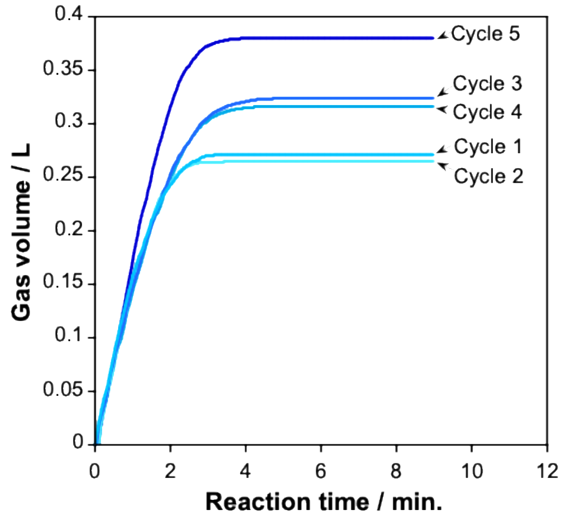

Figure S14. Regeneration of hydrogen by FADH after passage through the SACER6: Reaction conditions; FA aqueous solution after passing SACER6, 10 μmol of Cp*Ir-4DABP, and 80 ℃ of reaction temperature.

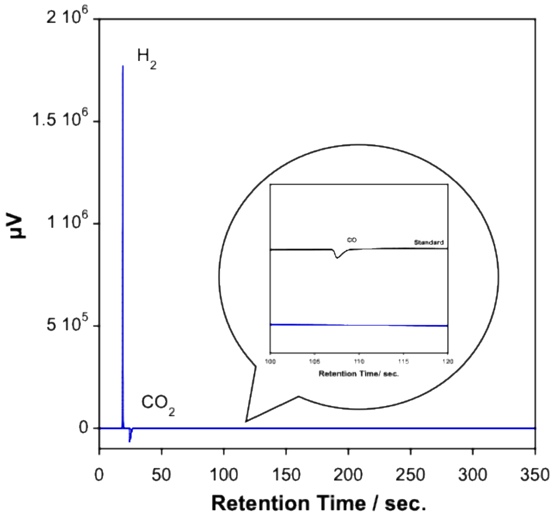

Figure S15. Gas composition analysis of generated gas from FA dehydrogenation (blue line) and the reference CO gases (black line).
